# Subcutaneous nodules accompanied by joint pain and erythema

**DOI:** 10.1016/j.jdcr.2025.02.008

**Published:** 2025-03-07

**Authors:** Frank Z. Jing, Alexander S. Hines, Michael Camilleri, Julio C. Sartori-Valinotti

**Affiliations:** a Department of Dermatology, Mayo Clinic, Rochester, Minnesota; b Division of Dermatopathology, Department of Dermatology, Mayo Clinic, Rochester, Minnesota

**Keywords:** pancreatic carcinoma, pancreatic panniculitis, pancreatitis, panniculitis, polyarthritis

## History

**Figure 1:**
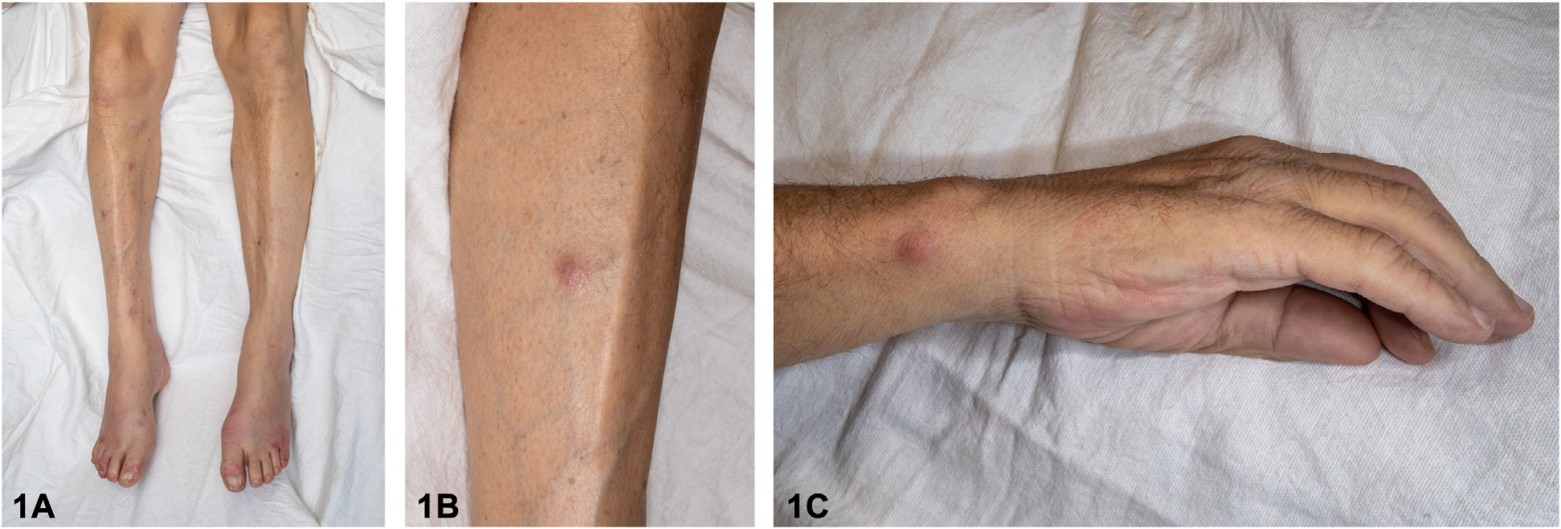

**Figure 2:**
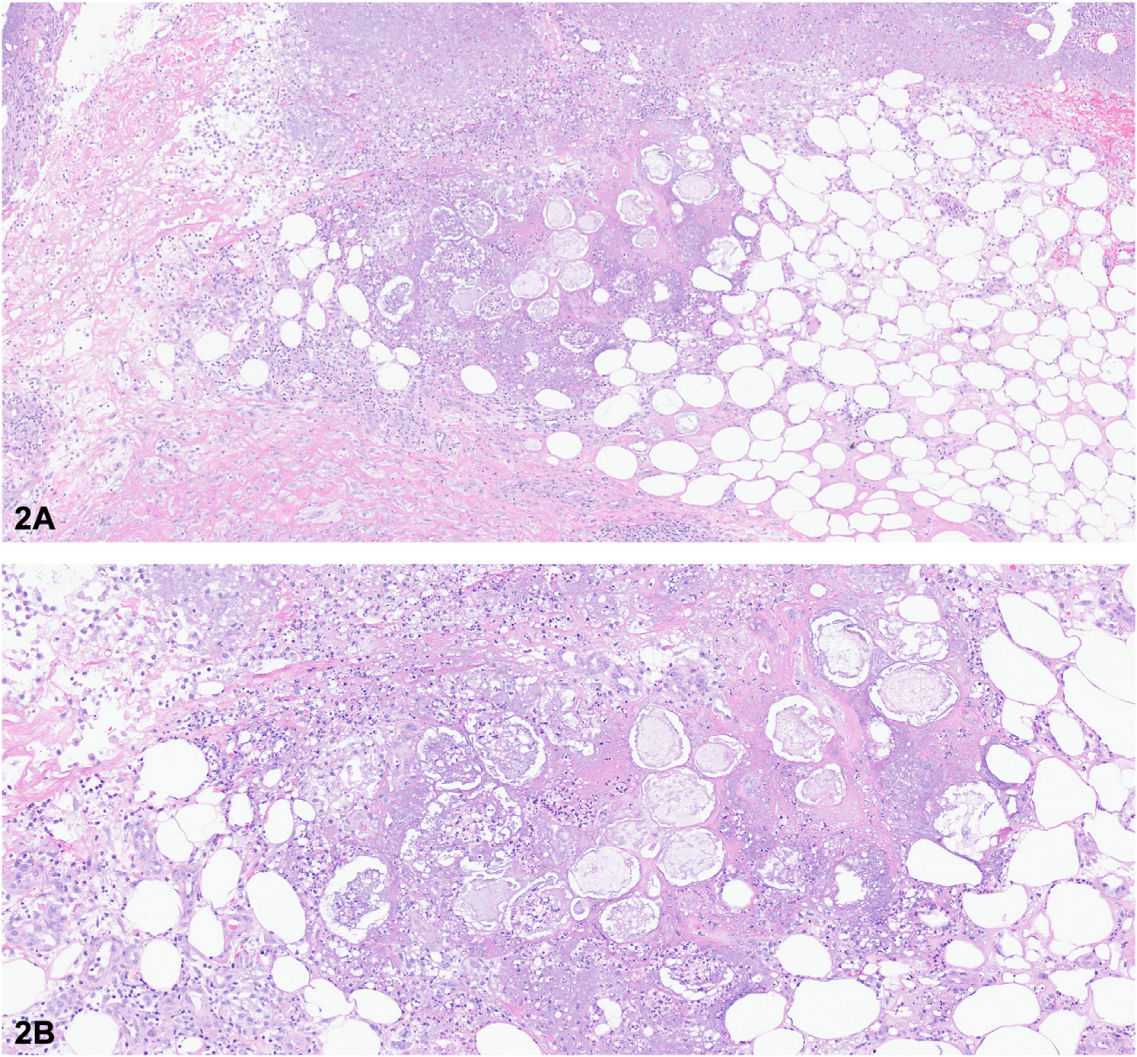

A 59-year-old man with history of gout, alcohol use disorder, and chronic pancreatitis presented with a 2-day history of erythema, swelling, and pain in his bilateral wrists, feet, and toes following a flare of chronic pancreatitis. Physical examination revealed erythema and swelling involving the wrists, ankles, and toes. He additionally had minimally tender 5 to 20-mm erythematous subcutaneous nodules on the radial aspect of the right wrist, bilateral anterior lower portion of the legs, and ankles (Fig 1, *A*-*C*). Laboratory workup revealed an elevated C-reactive protein of 159 mg/L (normal < 5 mg/L), leukocytosis with neutrophilic predominance at 10.9 × 10^9^/L, and an elevated lipase of 5555 U/L (normal 13-60 U/L). A computed tomography scan of the abdomen revealed peripancreatic stranding and fluid in the uncinate process. Magnetic resonance imaging of the left foot was suggestive of developing bone infarcts. A punch biopsy from a subcutaneous nodule on the left side of the lower extremity revealed lobular pannicular neutrophilic inflammation with fat necrosis consisting of ghost cells with calcium deposition (Fig 2, *A*, *B*). Acid-fast bacilli, fite, Grocott-Gömöri methenamine silver, and periodic acid–Schiff with diastase stains returned negative.

**Question 1: What is the diagnosis?**

A. Alpha-1 antitrypsin deficiency
B. Erythema nodosum
C. Pancreatitis, panniculitis, and polyarthritis syndrome (PPPS)
D. Polyarteritis nodosa
E. Sarcoidosis

**Answers:**

A. Alpha-1 antitrypsin deficiency – Incorrect. Although the histology of the subcutaneous nodules of alpha-1 antitrypsin deficiency displays lobular panniculitis with neutrophilic infiltrate, ghost cells would not be observed.
B. Erythema nodosum – Incorrect. Although the subcutaneous nodules of erythema nodosum are classically involving the lower extremities such as this patient, they are typically tender, and biopsy should demonstrate septal panniculitis instead of lobular panniculitis with ghost cells.
C. PPPS – Correct. This diagnosis accurately reflects the expected histologic findings of pancreatic panniculitis while also taking into account the extensive articular involvement and imaging suggestive of developing bone infarcts.^1^
D. Polyarteritis nodosa – Incorrect. Although polyarteritis nodosa can present with lower extremity subcutaneous nodules and arthralgias, the markedly elevated lipase levels and presence of ghost cells on histology do not support this diagnosis.
E. Sarcoidosis – Incorrect. Although sarcoidosis can present with arthralgias and lower extremity subcutaneous nodules resembling this case, the biopsy findings would likely demonstrate septal panniculitis. Moreover, the elevated lipase levels are not explained by a diagnosis of sarcoidosis.

**Question 2: What is the most frequent cause of PPPS**?

A. Alcohol
B. Cholelithiasis
C. Iatrogenic
D. Pancreas transplant
E. Pancreatic carcinoma

**Answers:**

A. Alcohol – Incorrect. Acute pancreatitis is the most common category of triggers for PPPS. However, alcohol is not the leading precipitating factor within this group. In a review by Seguí et al,^2^ alcohol was responsible for only 2 of 34 cases.
B. Cholelithiasis – Correct. In a review by Seguí et al,^2^ cholelithiasis precipitated 17 out of 34 cases, the most of any recorded triggering factor.
C. Iatrogenic – Incorrect. Although iatrogenic factors are a reported trigger for PPPS, they are not the most frequent cause overall.^2^
D. Pancreas transplant – Incorrect. Pancreas transplant is an uncommon precipitant of PPPS, accounting for only 1 out of 34 cases in a review by Seguí et al.^2^
E. Pancreatic carcinoma – Incorrect. Although pancreatic carcinoma is a more frequent trigger than chronic pancreatitis, it is less frequent than cholelithiasis as a precipitant of PPPS.^2^

**Questions 3: Which of the following is a true statement?**

A. Patients with PPPS precipitated by underlying pancreatic carcinoma generally have higher lipase levels compared with patients with acute pancreatitis
B. Intra-articular corticosteroid injections are effective in the management of articular pain in patients in PPPS
C. The majority of PPPS cases respond well to systemic steroids or nonsteroidal anti-inflammatory drugs
D. The most common type of pancreatic carcinoma associated with PPPS is pancreatic ductal adenocarcinoma
E. The Splendore-Hoeppli phenomenon is not observed in pancreatic panniculitis histology

**Answers:**

A. Patients with PPPS precipitated by underlying pancreatic carcinoma generally have higher lipase levels compared with patients with acute pancreatitis – Correct. Patients with pancreatic neoplasms are generally older, have higher lipase levels, and experience delayed diagnosis than those with underlying pancreatitis.^3^
B. Intra-articular corticosteroid injections are effective in the management of articular pain in patients in PPPS – Incorrect. A review of 28 patients who received pharmacologic treatments targeting articular and cutaneous manifestations, including systemic corticosteroids, nonsteroidal anti-inflammatory drugs, and intra-articular corticosteroid injections, revealed that 78.6% demonstrated poor response to therapy.^4^
C. The majority of PPPS cases respond well to systemic steroids or nonsteroidal anti-inflammatory drugs – Incorrect. Additional details are provided in the explanation for answer choice B.
D. The most common type of pancreatic carcinoma associated with PPPS is pancreatic ductal adenocarcinoma – Incorrect. The most common type of pancreatic carcinoma associated with PPPS is acinar cell carcinoma.^5^
E. The Splendore-Hoeppli phenomenon is not observed in pancreatic panniculitis histology – Incorrect. Although not typically associated with pancreatic panniculitis, the Splendore-Hoeppli phenomenon was identified in 9 out of 34 cases reported in a review by Seguí et al.^2^

## Conflicts of interest

None disclosed.
